# A CpG island methylator phenotype of colorectal cancer that is contiguous with conventional adenomas, but not serrated polyps

**DOI:** 10.3892/ol.2014.2430

**Published:** 2014-08-08

**Authors:** KOJI HOKAZONO, TAKASHI UEKI, KINUKO NAGAYOSHI, YASUNOBU NISHIOKA, TATSUNOBU HATAE, YUTAKA KOGA, MINAKO HIRAHASHI, YOSHINAO ODA, MASAO TANAKA

**Affiliations:** 1 Department of Surgery and Oncology, Graduate School of Medical Sciences, Kyushu University, Fukuoka, Fukuoka 812-8582, Japan; 2 Department of Anatomical Pathology, Graduate School of Medical Sciences, Kyushu University, Fukuoka, Fukuoka 812-8582, Japan

**Keywords:** CpG island methylator phenotype, colorectal cancer, methylation, promoter CpG island, serrated pathway

## Abstract

A subset of colorectal cancers (CRCs) harbor the CpG island methylator phenotype (CIMP), with concurrent multiple promoter hypermethylation of tumor-related genes. A serrated pathway in which CIMP is developed from serrated polyps is proposed. The present study characterized CIMP and morphologically examined precursor lesions of CIMP. In total, 104 CRCs treated between January 1996 and December 2004 were examined. Aberrant promoter methylation of 15 cancer-related genes was analyzed. CIMP status was classified according to the number of methylated genes and was correlated with the clinicopathological features, including the concomitant polyps in and around the tumors. The frequency of aberrant methylation in each CRC showed a bimodal pattern, and the CRCs were classified as CIMP-high (CIMP-H), CIMP-low (CIMP-L) and CIMP-negative (CIMP-N). CIMP-H was associated with aberrant methylation of *MLH1* (P=0.005) and with an improved recurrence-free survival (RFS) rate following curative resection compared with CIMP-L/N (five-year RFS rate, 93.8 vs. 67.1%; P=0.044), while CIMP-N tumors were associated with frequent distant metastases at diagnosis (P=0.023). No concomitant serrated lesions were present in the tumors, whereas conventional adenoma was contiguous with 11 (10.6%) of 104 CRCs, including four CIMP-H CRCs. CIMP-H was classified in CRCs by a novel CIMP marker panel and the presence of concomitant tumors revealed that certain CIMP-H CRCs may have arisen from conventional adenomas.

## Introduction

The presence of the CpG island (CGI) methylator phenotype (CIMP) in colorectal cancers (CRCs) has been supported by the fact that one group of CRCs has few methylated promoter CGIs and another group harbors simultaneous aberrant methylation of multiple promoter CGIs (1,2). CIMP-positive CRCs have distinct clinical and histological features, including a female predominance and proximal location, and show genetic characteristics, including frequent *KRAS*/*BRAF* mutations and microsatellite instability (MSI) (3,4). CIMP is initially defined using cancer-specific CIMP markers (*CDKN2A, MINT1, MINT2, MINT31* and *MLH1*) in CRCs (2), but in 2006, Weisenberger *et al* (5) challenged the application of these classic CIMP markers and insisted upon the efficacy of novel marker panels to endorse the CIMP as a distinctive molecular feature of CRCs. Although based on a systematic analysis of a large number of CRCs with aberrant methylation of numerous promoter CGIs, later studies failed to emulate the original results using the same markers selected by Weisenberger *et al* (4,6). No matter how the markers are selected, CIMP is certain to be involved in CRC development as the third molecular pathway, following chromosomal instability and MSI. Ogino *et al* showed that CIMP-positive CRC was a predictor of a low cancer-specific mortality rate in a large cohort study (4). By contrast, using different CIMP marker panels, this characteristic of CIMP-positivity was not observed in patients with stage III CRCs (7) or stage II/III CRCs treated by surgery alone (8). The response to 5-fluorouracil (5-FU)-based adjuvant chemotherapy in CIMP-positive CRC is also contradictory (9–11), although this therapy is essential to reduce the tumor recurrence of stage II or III CRC patients following curative resection.

Hypermethylation of promoter CGIs can prevent transcription of tumor suppressor or mismatch repair genes, such as MutL homolog 1 (*MLH1*), and occurs at an early stage of colorectal carcinogenesis. Methylation of promoter CGIs followed by transcriptional silencing of *MLH1* is present in ~70% of sporadic MSI CRCs (8,12,13). However, *MLH1* is usually included in CIMP marker sets of promoter CGIs, and up to 60% of CIMP-positive CRCs have aberrant methylation of *MLH1* (14). This may be one of the reasons for the clinical and pathological resemblance between CIMP-positive and MSI CRCs. The high frequency of serrated polyps with *MLH1* gene promoter methylation in individuals with MSI CRC suggests the presence of a serrated pathway in colorectal carcinogenesis (15). More recently, genetic and epigenetic profiles of a variety of colorectal polyps have demonstrated that sessile serrated adenomas/polyps may be precursor lesions for MSI CRCs and follow the CIMP pathway (16). Since a considerable fraction of advanced CRCs in the adenoma-adenocarcinoma sequence had a remnant adenomatous element within the tumors and coexisting extralesional adenomas (17), it is important to examine whether CIMP-positive CRCs have similar morphological characteristics regarding serrated polyps.

In the present study, a series of CRCs were retrospectively examined for aberrant methylation using an alternative panel of promoter CGIs of cancer-related genes. The panel consisted of promoter CGIs of tumor suppressor genes (*p16, GATA5, TSLC1, HLTF* and *ID4*), DNA repair genes (*MGMT*), metastases suppressor genes (*TIMP3, CDH4* and *CDH13*), angiogenesis inhibitors (*TSP1*) and genes with apoptosis-related properties (*HRK, CACNA1G* and *RSASF1A*). The majority of these genes are not involved as classical or novel CIMP markers of CRC (2,5,14). The panel also contained two novel genes that were originally cloned in pancreatic cancer, which were methylated in the cancer cells, but not in the normal pancreas or colonic mucosa (18). The CIMP was defined by comparing the observed distribution of CRCs by the number of aberrant methylations of these genes with the calculated distribution. The CIMP status was correlated with the methylation status of *MLH1* and with clinicopathological parameters, with particular reference to neighboring lesions, such as conventional adenoma and serrated lesions, in and around tumors.

## Materials and methods

### Patient population and DNA preparation

Neoplastic specimens were collected from consecutive patients who underwent CRC resection at Kyushu University Hospital (Fukuoka, Japan) between 1996 and 2004. From these tumor specimens, 104 CRC frozen tissues were used and the frozen tissue of 15 corresponding normal mucosae were also collected. Clinical data and the patient status at the last follow-up were obtained from medical records. Informed consent to harvest the tissue for the studies was obtained from all patients, and the Kyushu University Hospital Human Research Ethics Committee approved the study. Genomic DNA was prepared from cryostat sections of the frozen cancer tissue and corresponding normal mucosa specimens and was extracted by QIAamp DNA Mini kit (Qiagen, Hilden, Germany). Hematoxylin and eosin-stained sections of formalin-fixed and paraffin-embedded surgical specimens were evaluated to determine tumor differentiation and stage. All polyps present in the specimen were also sectioned and prepared for histological examination.

### Bisulfite modification and methylation-specific polymerase chain reaction (MSP) assay

The methylation status of each gene was verified by MSP, as described by Herman *et al* (19). Genomic DNA from the cancer tissue and the normal mucosa was treated with bisulfite for 16 h at 50°C to convert unmethylated cytosine to thymine. Polymerase chain reaction (PCR) primers for each gene were designed to be specific for the methylated sequence and the promoter region of each gene. Three to six CpG sites were included in each primer pair to achieve optimal specificity for the determination of methylation. MSP was carried out on 1 μl bisulfite-treated DNA with the following amplification conditions: 95°C for 5 min, followed by 40 cycles of 94°C for 30 sec, annealing for 30 sec and 72°C for 30 sec, with a final extension at 72°C for 5 min. All PCRs were performed with CpGenome Universal Methylated DNA (Chemicon International, Temecula, CA, USA) as a positive control for methylated alleles and with no DNA as a negative control. The primer sequences and the specific annealing temperatures for the 15 genes and *MLH1* are shown in Table I. PCR products (5 μl) were separated by 3% agarose gel electrophoresis and visualized under ultraviolet illumination, following ethidium bromide staining. The presence of PCR products indicated the presence of methylated template sequences in the original genomic DNA.

### Statistical analysis

The primary variable in this study was the distribution of carcinomas falling into each classification of the number of aberrantly methylated genes. The observed distribution of the 104 CRCs was compared with the expected distribution by χ^2^ test (goodness-of-fit test) under the assumptions that promoter methylation of each gene occurred randomly and was distributed equally in the carcinomas. The association between CIMP status and clinicopathological parameters was assessed by Fisher’s exact test, analysis of variance or Mann-Whitney U test. Event time distributions for overall survival (OS) and relapse-free survival (RFS) of our 104 CRC patients were estimated with the Kaplan-Meier method. Hazard ratios (HRs) of tumor-relapse, according to the clinicopathological features and the CIMP status in tumors, were analyzed by Cox proportional hazard models. All P-values were two-sided, and P<0.05 was considered to indicate a statistically significant difference.

## Results

### Classification of CIMP and methylation of each gene in CRCs

**Figure 1:**
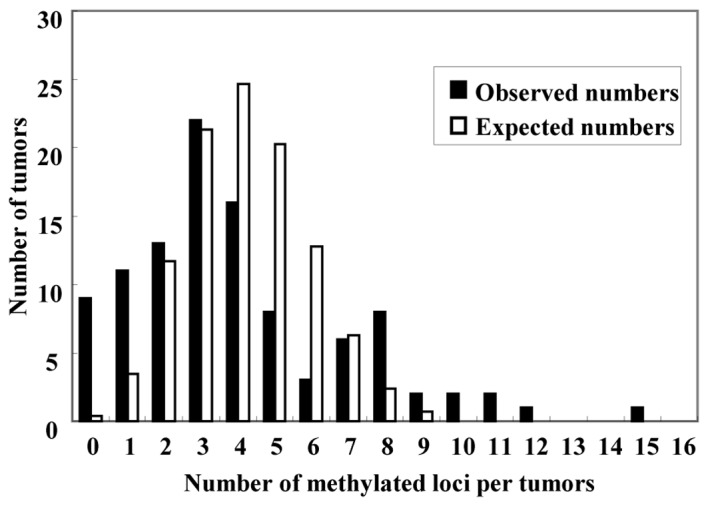
Distribution of the number of tumors and methylated loci per tumor. The expected number is represented by a white bar, and the observed number by a black bar. The observed distribution of carcinomas with each number of aberrantly methylated genes appeared divided into two groups between the high and low methylated groups. With the CpG island methylator phenotype (CIMP) classification used, there was a significant difference between the expected and observed tumor distribution (goodness-of-fit test, P<0.001). Carcinomas with methylation of ≥7 of the 15 genes was classified as CIMP-high, methylation of 1–6 genes as CIMP-low, and without any methylation as CIMP-negative.

**Figure 2:**
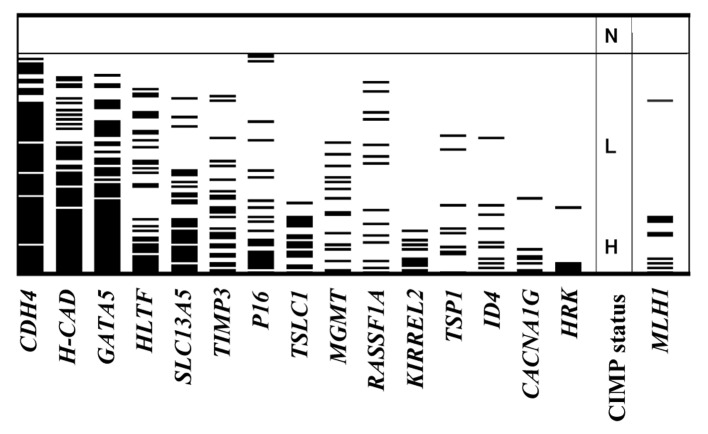
Summary of methylation of 15 promoter CpG islands in 104 colorectal cancers. Gray bars indicate methylation of each gene. CpG island methylator phenotype (CIMP) status and MutL homolog 1 (*MLH1*) methylation shown by gray bars are specified on the right of the methylation data map.

The number of aberrantly methylated genes in each CRC ranged between zero and 14. The expected distribution of carcinomas having each number of aberrantly methylated genes among 104 CRCs was calculated, assuming that hypermethylation of the 15 genes occurred independently and was spread randomly across the 104 CRCs. The expected distribution followed a unimodal pattern, with the largest number of carcinomas having four methylated genes (Fig. 1, white bars). In the expected distribution, ten CRCs (9.6% of the 104 CRCs) were predicted to have seven or more methylated genes, and the number of CRCs without any methylated genes was zero (0% of the 104 CRCs). The observed distribution of carcinomas having each number of aberrantly methylated genes did not follow the expected distribution (Fig. 1, black bars). Carcinomas with methylation of seven or more of the 15 genes were classified as CIMP-high (CIMP-H), carcinomas with methylation of one to six genes were classified as CIMP-low (CIMP-L) and carcinomas without methylation were classed as CIMP-negative (CIMP-N). There were 19 (18.3%) CIMP-H CRCs, 76 (73.1%) CIMP-L CRCs and 9 (8.7%) CIMP-N CRCs. The observed distribution of methylated genes in each group differed significantly from the expected distribution (P<0.001), thus, methylation of these genes did not happen at random in the 104 CRCs. Methylation of each gene and the CIMP status are summarized in Fig. 2. The frequency of hypermethylation of the 15 genes in the 104 CRCs ranged from 5.8% (*HRK*) to 77.9% (*CDH4*), whereas methylated templates were not detected in the 15 normal colonic mucosae.

### CIMP status, clinicopathological parameters and MLH1 methylation

The association between CIMP status, the clinicopathological features and *MLH1* methylation are shown in Table II. There was no significant correlation between the CIMP status and the parameters among the 104 patients with respect to age, gender, tumor size, histological tumor grade or tumor stage. In total, 12 patients exhibited distant metastases, the majority of which were liver metastases, and three patients presented with peritoneal metastases at diagnosis. *MLH1* methylation was detected in 10 (9.6%) of 104 CRCs. Six of these were classified as CIMP-H (31.6% of 19 CIMP-H tumors) and four were CIMP-L (5.3% of 76 CIMP-L tumors), but none of the nine CIMP-N tumors exhibited *MLH1* methylation (P=0.005). The patients with CIMP-N CRCs had more frequent distant metastases compared with those with CIMP-H/L tumors (44.4, 15.8 and 10.5%, respectively, P=0.023). 5-FU-based chemotherapy was post-operatively performed in 63.2% of patients with CIMP-H CRCs, 64.5% of those with CIMP-L and 77.8% of those with CIMP-N. Within the median follow-up time of 60 months, two (40.0%) out of five patients with stage 0-III CIMP-N and 22 (32.4%) out of 68 patients with stage 0-III CIMP-L developed tumor recurrence following curative resection, while only one (6.3%) out of 16 patients with stage 0-III CIMP-H tumors developed recurrence (P=0.093).

**Figure 3:**
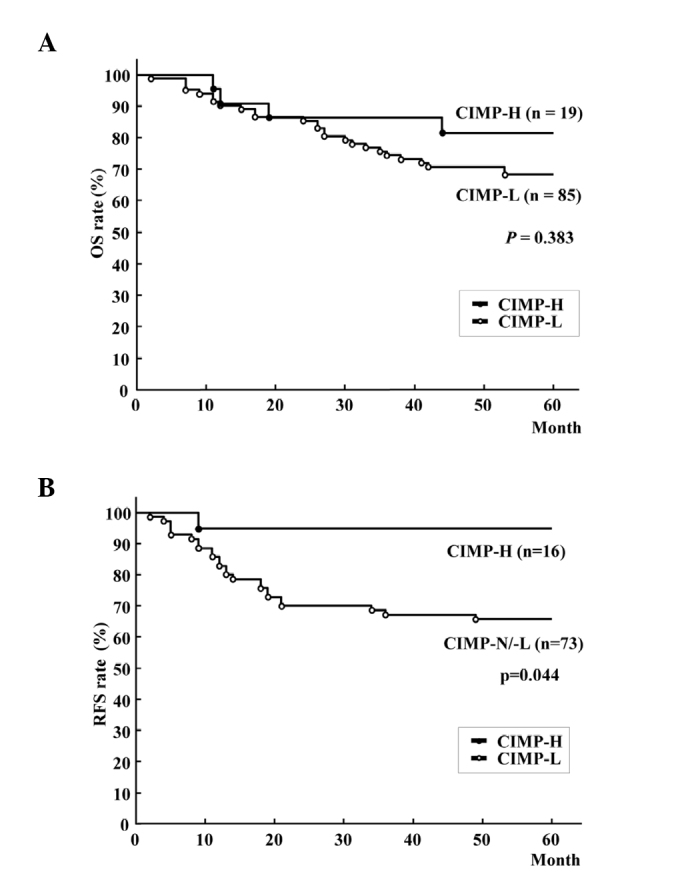
Kaplan-Meier survival curves for (A) OS rate of all patients and (B) RFS of patients following curative resection of colorectal cancer. OS rate was not significantly different between patients with CIMP-H and CIMP-L/-N (five-year OS rate, 79.0% vs. 68.2%; log-rank test, P=0.383), while patients with CIMP-H tumors had better RFS rates than those with CIMP-N/L tumors (five-year RFS rate, 93.8 vs. 67.1%; log-rank test, P=0.044). CIMP-H, CpG island methylator phenotype-high; CIMP-L, CIMP-low; CIMP-N, CIMP-negative; RFS, recurrence-free survival; OS, overall survival.

The rate of tumor recurrence in the CIMP-N and CIMP-L tumors was similar; therefore, 104 CRCs were divided into two groups, CIMP-L/N and CIMP-H, for further survival time analysis. Kaplan-Meier survival curves representing the OS rates of all patients and the RFS rate of 89 patients with stage 0-III tumors, according to CIMP status, are shown in Fig. 3A and B. Patients with CIMP-H CRCs exhibited a significantly improved RFS rate compared with those with CIMP-L/N CRCs (Fig. 3B; five-year RFS rate, 93.8 vs. 67.1%; log-rank test, P=0.044), although there was no significant difference in OS rate (Fig. 3A; five-year OS rate, 79.0 vs. 68.2%; P=0.383). Cox regression univariate analysis revealed that CIMP-H was a better prognostic indicator for tumor recurrence following curative resection [HR, 0.167; 95% confidence interval (CI), 0.001–0.789], with stage 0-II tumors, an absence of lymphatic and venous invasion and *MLH1* methylation as better prognostic factors (Table III). Although the multivariate analysis revealed that tumor stage 0-II was a significantly better prognostic factor (Table III), the HR of the multivariate analysis for tumor recurrence in CIMP-H tumors was consistently low in stage 0-II (HR, <0.001; 95% CI, 0.000–2.281) and in stage III (HR, 0.455; 95% CI, 0.003–2.228) tumors.

### Coexistent lesions within tumors and in the normal mucosae surrounding CRC

In total, 11 (10.6%) of 104 CRCs presented with neighboring conventional adenoma, but no CRC had adjacent serrated lesions. Among the CRCs with adenoma, two were T0, three were T1, one was T2 and five were T3. Four (21.1%) out of 19 CIMP-H CRCs presented with coexistent adenomas, together with six (7.9%) out of 76 CIMP-L and one (11.1%) out of nine CIMP-N CRCs. Two out of four CIMP-H CRCs with adenoma were located in the right colon and the others were in the left colon/rectum; two were T1 and two were T3 tumors. One had *MLH1* promoter hypermethylation and the remaining three had no *MLH1* hypermethylation. Among the 104 CRC resections, serrated lesions, including five hyperplastic polyps and one serrated adenoma, were present in the normal mucosae around the tumors of four specimens, while conventional adenomas were detected in 25 specimens. In the tumor specimens containing serrated lesions, two CRCs were located in the right colon and two in the left colon/rectum. The serrated lesions were present in one specimen with CIMP-H, in two with CIMP-L and in one with CIMP-N CRC, while adenomatous lesions were distributed in five specimens with CIMP-H, 17 with CIMP-L and three with CIMP-N CRC.

## Discussion

In the present study, according to the number of hypermethylations of 15 promoter CGIs, an almost bimodal distribution of CRCs indicated the presence of the distinct subclass of CRC, termed CIMP-H, which is recognized by an accumulation of hypermethylation of promoter CGIs. In other words, tumor-specific aberrant methylation in promoter CGIs may assemble itself in CIMP-H and randomly occur in remaining CRCs. Although this bimodal distribution in tumors has been demonstrated using several gene marker panels (5,14), Yamashita *et al* (20) doubted the presence of CIMP, claiming that tumor-specific somatic hypermethylation of six genes (*MLH1*, *p16, p14, MGMT, APC* and *CDH1*) was an age-dependent feature and that the distribution of the number of tumors harboring their markers was normal (20). This inconsistency could have been due to the different marker panels used in each study. For instance, *APC* gene methylation has been inversely linked to classical CIMP-H CRCs (21). Weisenberger *et al* demonstrated the bimodal distribution of tumors using 14 novel CIMP markers, but the histogram of the methylation frequency of the five classic CIMP markers showed only one peak (5). In the present study, all 15 markers, with the exception of one (*CACNA1G*), differed from Weisenberger’s 14 markers, while the histograms resembled each other. Furthermore, Ogino *et al* also identified that the CIMP classification error decreased along with an increasing number of markers from one to seven (14). Thus, in addition to the selection of markers, the number of promoter CGIs examined is crucial for the detection of CIMP. The mechanism of this epigenetic instability is unresolved, thus, the definition of CIMP cannot be faultless and depends on the distribution of the methylation frequency of selected markers.

Following the identification of concurrent methylation of several classic CIMP markers in hyperplastic polyposis, large hyperplastic polyps and serrated adenomas (22,23), the serrated lesions, particularly sessile serrated adenomas/polyps, have been described as conceivable precursors of the serrated pathway to CIMP-H CRCs (16,24). In the present study, serrated lesions contiguous with CRC were not found in any of the CIMP-H tumors. Although this may account for the notion that the ancestor lesion would be replaced by an aggressive successor, >20% of CIMP-H CRCs had concomitant adenomatous lesions that indicated the adenoma-carcinoma pathway of their tumorigenesis. The definition of CIMP-H in the present study was different from the classic or novel CIMP classification, thus the present CIMP-H CRCs may differ from those on the advocated serrated pathway. Jass (25) proposed that one of the molecular subtypes of CRCs that is characterized by CIMP-L, *KRAS* mutation and microsatellite stable/MSI-L, originates from adenoma or serrated polyps. Although the present study did not examine this molecular discrimination, certain CIMP-H CRCs that have coexistent adenomas can be classified in this subtype. These CIMP-H CRCs may arise from serrated components of mixed hyperplastic and adenomatous polyps, but these polyps are rare. Only one CIMP-H CRC specimen exhibited serrated lesions around the tumor, and each CIMP subtype did not vary in the frequency of serrated lesions in the colonic mucosa around the tumors. Thus, the results failed to support the serrated pathway in CIMP-H CRC tumorigenesis, but suggested that a certain fraction of CRCs showing promoter hypermethylation of multiple cancer-related genes is derived from conventional adenoma.

In univariate analysis, the patients with CIMP-H tumors exhibited a significantly improved disease-free survival rate compared with those with CIMP-L/N tumors following curative resection. Similar results have been obtained from a large cohort (4), but this prognostic advantage of CIMP-H is often challenged (7,8,26). Any attempt to involve *MLH1* in the CIMP marker panel (14) and to correlate CIMP-H with *BRAF* mutation (5) would confuse the aforementioned argument further. *MLH1* methylation followed by MSI-high (MSI-H) is known to be associated with a good prognosis in patients with sporadic CRC (27–29), while *BRAF* mutation is one of the genetic markers of a shorter survival time (4,30). Similarly, this controversy over the prognosis of patients with CIMP-H tumors is occasionally explained by the presence of the CIMP subtypes harboring various prognostic features, CIMP-H/MSI-H with frequent *BRAF* mutation and CIMP-H/MSS with occasional *BRAF,* but a dominant *KRAS* mutation (31). Patients with MSI-H CRCs do not benefit from 5-FU-based adjuvant chemotherapy (32,33). Although CIMP-H was associated with *MLH1* methylation in the present study, *MLH1* methylation was not an independent factor for an improved RFS rate. Additionally, 68.4% of CIMP-H tumors had no *MLH1* methylation and 63.2% of patients with CIMP-H tumors received post-operative 5-FU-based adjuvant chemotherapy in the present study. Thus, the improved RFS rates of patients with CIMP-H tumors was not solely a result of MSI-H following *MLH1* methylation. Iacopetta *et al* demonstrated that CIMP-H was a predictor of the survival benefit from 5-FU-based chemotherapy in CRC patients (9). Therefore, this prognostic advantage of patients with CIMP-H CRCs could be augmented by 5-FU-based adjuvant chemotherapy. However, 5-FU-based adjuvant chemotherapy was recently shown not to improve, but to worsen the disease-free survival of patients with stage II or III CIMP-positive CRC (34). The criteria of CIMP, the proportion of MLH methylation in CIMP-H tumors and the number of patients receiving adjuvant chemotherapy largely differed between these studies. Thus, a large cohort study based on universal CIMP consent using coherent CIMP markers is required to resolve this critical issue.

Little is known about the CRCs that are without methylation of any promoter CGIs; named CIMP-N in the present study. The absence of aberrant methylation of any promoter CGIs in these patients confers possible global hypomethylation, which has been often associated with chromosomal instability in CRC (35,36). Instead, an inverse association between CIMP-H and chromosomal instability has been shown (37,38). Similar to the association between the shorter survival time and *LINE-1* hypomethylation among CRC patients (39), frequent metastases at the time of diagnosis in patients with CIMP-N CRC suggest that CIMP-N tumors are more aggressive than CIMP-L/H tumors. In the present study, the histogram of the CIMP-L/N CRCs was almost a Gaussian distribution and the number of tumors in this subset was small, therefore, the meaning of this phenotype in CRCs remains uncertain.

There were certain limitations to this study. Firstly, *BRAF* and *KRAS* were not sequenced. Although the *BRAF* mutation is one of the genetic traits of CIMP-H CRCs, the *BRAF* mutation in CIMP has varied in each study, ranging from 21.6% using a classic CIMP marker set (26), to 73% using novel markers (5). The mechanism connecting the *BRAF* mutation and aberrant methylation of promoter CGIs remains unclear, and the importance of this mutation to predict prognosis was not proven in recent larger studies involving >1,000 CRC patients (27,40). Additionally, MSI status was not examined in the present study. Finally, this was a single-institution retrospective study, and the numbers of patients and genes examined were not sufficient to allow definitive conclusions.

The panel of promoter CGIs in this study included *KIRREL2* and *SLC13A5*, which have previously been identified as the differentially-methylated CGIs in pancreatic cancer and cloned using methylated CpG island amplification-representational difference analysis from pancreatic cancer cell lines (18). Cancer-specific methylation of these CGIs, loss of expression of these genes in CRC cell lines that had hypermethylation of these promoter CGIs and restoration of their expression by 5-aza-2-deoxycytidine treatment (data not shown) suggest possible involvement of promoter methylation of these genes in colorectal carcinogenesis. For example, *SLC13A5*, a member of the solute carrier (SLC) families and a Na^+^/sulfate/selenate/thiosulfate/carboxylate symporter (41), is one of the hallmarks of CIMP in renal cell carcinoma (42). *SLC13A5* is differentially methylated between glioblastoma and normal brain tissue, as shown by whole-genome integrative analysis (43). Certain SLC family members increase chemosensitivity against anticancer drugs by mediating the cellular uptake of hydrophilic drugs (44). One of the sodium transporter families also has tumor suppressor activity, and aberrant methylation of promoter CGI is detected in aberrant crypt foci, which is considered to be the initial lesion of the serrated adenoma-carcinoma pathway (45). Thus, future studies on the novel target gene for aberrant promoter methylation would shed light on our understanding of cancer epigenetics and the carcinogenesis of CRC.

## Figures and Tables

**Table I:** Methylated primer sequences and annealing temperatures used in methylation-specific polymerase chain reaction.

Gene	F/R	Sequences	Temperature, °C	Size, bp
*p16*	F	5′-TAATAGTATTTTTTTCGAGTATTC-3′	54	123
	R	5′-TTCTTCCTCCGATACTAACG-3′		
*hMLH1*	F	5′-TAATAGGAAGAGCGGATAGC-3′	54	106
	R	5′-TCTATAAATTACAAATCTCTTCG-3′		
*TIMP3*	F	5′-GGGTCGATGAGGTAATGC-3′	64	116
	R	5′-TACGCCGCTACCTAAACG-3′		
*MGMT*	F	5′-GTTTTAGATTTCGTTTTACGTC-3′	54	145
	R	5′-CCAAATCGCAAACGATACG-3′		
*TSP1*	F	5′-GAAAGTTTTTGCGTTATTTCGC-3′	64	130
	R	5′-CTTAACGCACACGAACTCG-3′		
*CACNA1G*	F	5′-TTTTAGATTCGGTTTTAGTTGC-3′	54	140
	R	5′-AACTCCGATTACCGAATTCG-3′		
*CDH4*	F	5′-GTTTTCGGTGTCGGGTATC-3′	66	105
	R	5′-CGACAACTTACCCGAAACG-3′		
*H-CAD*	F	5′-TTCGCGGGGTTCGTTTTTC-3′	67	147
	R	5′-AATAAATCAACAACAACATCACG-3′		
*GATA5*	F	5′-TTCGGGTCGTTGAGGTTTC-3′	64	140
	R	5′-CAAAATCACGTAACTCTACG-3′		
*RASSF1A*	F	5′-CGAGAGCGCGTTTAGTTTC-3′	58	103
	R	5′-CAAAATCCAAACTAAACGACG-3′		
*HLTF*	F	5′-CGTTTCGTTGTTATTTAAAGAC-3′	60	132
	R	5′-CCGCAAACACCGCAATCG-3′		
*HRK*	F	5′-AATTTCGCGTTTTTTAGTTGTC-3′	54	115
	R	5′-GAAAAAAAAAATTACATCATCCG-3′		
*KIRREL2*	F	5′-TTGGGGGCGTTTATTCGTC-3′	62	105
	R	5′-GCCCCCCGAAAACTCCG-3′		
*SLC13A5*	F	5′-GTTTAGCGTCGAGGTTATC-3′	67	137
	R	5′-TACGAAACGAAATTATCACCG-3′		
*ID4*	F	5′-ATTTTTCGTTTTTTAGTATCGTTC-3′	62	104
	R	5′-ACGCGCGAACCGAATCG-3′		
*TSLC1*	F	5′-TAATCGTTGTATTAGATCGAC-3′	60	103
	R	5′-TAAATTTACAACGTCTAATTCG-3′		

F, forward primers; R, reverse primers; *hMLH1*, human MutL homolog 1.

**Table II:** Association between the CIMP and the clinicopathological features of 104 colorectal cancers.

Features	Total	CIMP-N	CIMP-L	CIMP-H	P-value^a^
No. of patients	104 (100.0)	9 (8.7)	76 (73.1)	19 (18.3)	
Mean age, years	63.4	60.7	62.9	66.6	0.317
Gender, n (%)					
Male	51 (49.0)	5 (55.6)	35 (46.1)	10 (52.6)	
Female	53 (51.0)	4 (44.4)	41 (53.8)	9 (47.4)	0.471
Tumor location, n (%)					
Proximal	42 (40.4)	1 (11.1)	32 (42.1)	9 (47.4)	
Distal	62 (59.6)	8 (88.9)	44 (57.9)	10 (52.6)	0.118
Mean tumour size, mm	48.2	47.7	49.7	43.7	0.466
Histology, n (%)					
Differentiated	90 (86.5)	7 (77.8)	67 (91.3)	16 (84.2)	
Undifferentiated	14 (13.5)	2 (22.2)	9 (8.7)	3 (15.8)	0.680
Lymphatic invasion, n (%)					
Negative	51 (49.0)	5 (55.6)	38 (50.0)	8 (42.1)	
Positive	53 (51.0)	4 (44.4)	38 (50.0)	11 (58.9)	0.760
Venous invasion, n (%)					
Negative	66 (57.4)	5 (55.6)	47 (60.3)	14 (73.7)	
Positive	38 (42.6)	4 (44.4)	29 (39.7)	5 (26.3)	0.543
Tumor stage, n (%)					
0	2 (1.9)	0 (0.0)	2 (2.6)	0 (0.0)	
I	16 (15.4)	1 (11.1)	12 (15.8)	3 (15.8)	
II	32 (30.8)	3 (33.3)	21 (20.2)	8 (42.1)	
III	39 (37.5)	1 (11.1)	33 (43.4)	5 (26.3)	
IV	15 (14.4)	4 (44.4)	8 (10.5)	3 (15.8)	0.227
Distant metastases at diagnosis, n (%)					
Negative	89 (85.6)	5 (55.6)	68 (89.5)	16 (84.2)	
Positive	15 (14.4)	4 (44.4)	8 (10.5)	3 (15.8)	0.023
Postoperative chemotherapy, n (%)					
No	35 (33.7)	2 (22.2)	26 (34.2)	7 (36.8)	
Yes	69 (66.3)	7 (77.8)	50 (65.8)	12 (63.2)	0.719
Tumor recurrence^b^, n (%)					
Negative	64 (71.9)	3 (60.0)	46 (61.6)	15 (93.7)	
Positive	25 (28.1)	2 (40.0)	22 (32.4)	1 (6.3)	0.093
*MLH1* methylation, n (%)					
−	94 (90.4)	9 (100.0)	72 (94.7)	13(68.4)	
+	10 (9.6)	0 (0.0)	4 (5.3)	6 (31.6)	0.005

a Assessed by analysis of variance or Kruskal-Wallis test.

b Stage 0-III tumors.

CIMP, CpG island methylator phenotype; CIMP-L, CIMP-low; CIMP-H, CIMP-high; CIMP-N, CIMP-negative, *MLH1,* MutL homolog 1; NS, not significant.

**Table III:** Univariate and multivariate analysis of risk factors for recurrence-free survival in stage 0-III colorectal cancer patients.

	Univariate analysis			Multivariate analysis		
		
Factor	HR	95% CI	P-value	HR	95%	CI P-value
Age, years (>60/≤60)	0.639	0.287–1.409	0.264			
Gender (male/female)	0.541	0.229–1.120	0.132			
Tumor location (proximal/distal)	0.683	0.278–1.536	0.364			
Tumor size, mm (<44/≥44)	0.961	0.435–2.134	0.919			
Histology (diff/undiff)	0.872	0.302–3.686	0.827			
Depth of tumor (T0–2/T3, T4)	0.416	0.098–1.201	0.112			
Tumor stage (stage 0-II/III)	0.247	0.096–0.568	0.001	0.395	0.146–0.969	0.042
Pathological lymphatic invasion (negative/positive)	0.448	0.189–0.994	0.048	0.527	0.220–1.187	0.123
Pathological venous invasion (negative/positive)	0.305	0.135–0.674	0.004	0.503	0.212–1.156	0.106
Post-operative chemotherapy (yes/no)	0.722	0.295–1.624	0.440			
CIMP status (CIMP-H/L/N)	0.167	0.001–0.789	0.019	0.292	0.016–1.421	0.149
*MLH1* methylation (positive/negative)	<0.001	0.601–0.601	0.013	<0.001	<0.001–1.888	0.141

Diff, differentiated; undiff, undifferentiated; CI, confidence interval; CIMP, CpG island methylator phenotype; CIMP-H, CIMP-high; CIMP-L, CIMP-low; CIMP-N, CIMP-negative; *MLH1*, MutL homolog 1.
